# Supplementary material for: Bimodal ionic photomemristor based on a high-temperature oxide superconductor/semiconductor junction
Source: Nat Commun. 2023 May 25;14:3010. doi: 10.1038/s41467-023-38608-0 (PMC10212962; doi:10.1038/s41467-023-38608-0)
Supplement: Supplementary file 1 — Supplementary Information [file 41467_2023_38608_MOESM1_ESM.pdf]

Supplementary information for

Bimodal ionic photo-memristor based on a high-temperature oxide superconductor/semiconductor junction

Ralph El Hage[1]. Vincent Humbert[1], Victor Rouco[1], Gabriel Sánchez-Santolino[2], Aurelien Lagarrigue[1], Kevin Seurre[1], S. Carreira[1], Anke Sander[1], Jérôme Charliac[3], Salvatore Mesoraca[1], Juan Trastoy[1], Javier Briatico[1], Jacobo Santamaría[1,2] & Javier E. Villegas[1,*]

[1]*Unité Mixte de Physique, CNRS, Thales, Université Paris Saclay, 91767 Palaiseau, France*

[2]*GFMC, Dpto. Física de Materiales. Universidad de Ciencias Físicas, Universidad Complutense de Madrid, 28040 Madrid, Spain*

[3]*Laboratoire de Physique des Interfaces et des Couches Minces (UMR7647), CNRS, Ecole Polytechnique, 91128 Palaiseau Cedex, France*

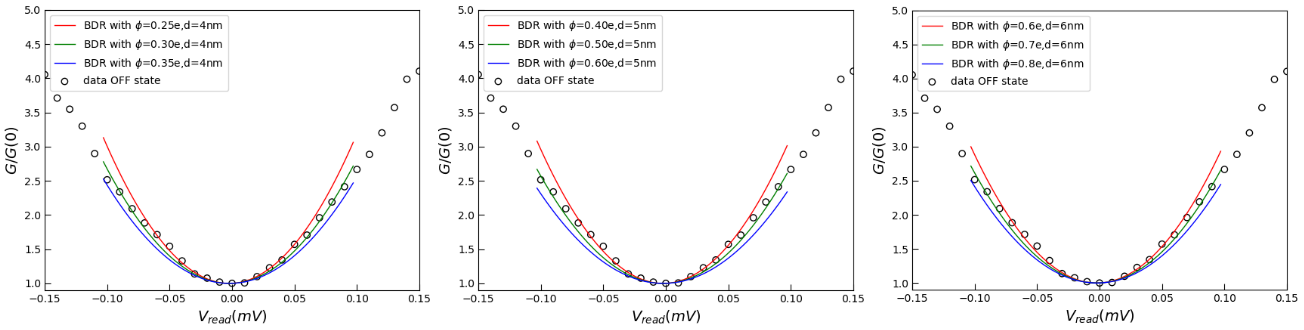

Figure S1: BDR analysis of the conductance. (scatter symbols) differential conductance (normalized to the zero-bias value) vs. bias measured for and YBCO/ITO junction in the normal state (T =100 K). The lines are the best fits using the Brinkman, Dynes, and Rowell (BDR) model for electron tunnelling, from which we can extract quantitative information about the tunnel barrier. Within this model, the conductance depends on the applied voltage through the following expression:

$$\frac{G(V)}{G(0)} = 1 - \left(\frac{A_0 \Delta\phi}{16\,\phi^{\frac{3}{2}}}\right) eV + \left(\frac{9\,A_0^2}{128\,\phi}\right) (eV)^2 \,,$$

where $\Delta\phi$ is the barrier asymmetry, ϕ is the average barrier height and $A_0 = \frac{4(2m)^{\frac{1}{2}}d}{3\hbar}$ with m the electron mass, and d the barrier thickness. The BDR fits presented in the figure have been obtained using the parameters shown in the inset, which yield $d = 5 \pm 1$ nm and $\phi = 0.5 \pm 0.2$ eV, with an asymmetry $\Delta\phi$ = 10 %. The barrier thickness d estimated from the fits is comparable with the thickness of the space charge layer of the *p*-*n* junction expected at the oxygen-depleted YBCO-ITO interface, $W \lesssim$ 5 nm. This is estimated considering[1] $W = \sqrt{(N_A + N_D / N_A N_D)\; 2\varepsilon_s V_{bi}/e}$ where $N_A \sim 8\ 10^{19}$ cm^{-3} and $N_D \sim 5\ 10^{20}$ cm^{-3} are respectively the carrier densities in oxygen-depleted YBCO[2] and in ITO[3], and an upper limit for built-in voltage $V_{bi} < 1.5\ V$ can be inferred from the difference between work functions.

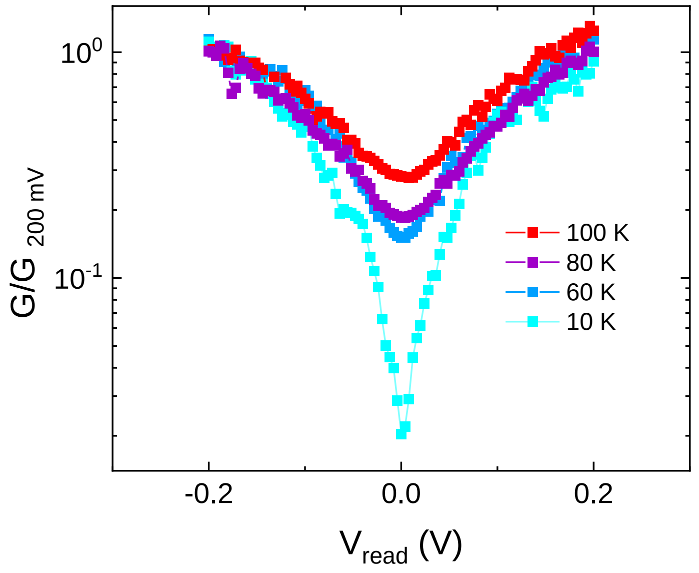

Figure S2: Temperature dependence of the conductance showing superconducting effects. Differential conductance *vs.* bias of an ITO/YBCO junction in the ON state, measured for different temperatures as indicated by the legend. A zero-bias conductance dip that strongly deepens as the temperature is decreased below the superconducting transition ($T_C \sim 90$ K) is observed. This behaviour was found earlier in MoSi/YBCO junctions[4] and is as expected for electron tunnelling into a superconductor[5]: as the temperature is decreased across the superconducting transition T_C, a gap opens around the Fermi energy, and electrons can only tunnel into quasiparticle excitations in the superconductor. Because the quasiparticle population diminishes as the temperature is decreased below T_C, the conductance around zero-bias (below the superconducting gap) decreases. This explains[4] the downturn (departure from the high-temperature trend) observed in the conductance vs. temperature curves displayed in the inset of the manuscript's Fig. 1b.

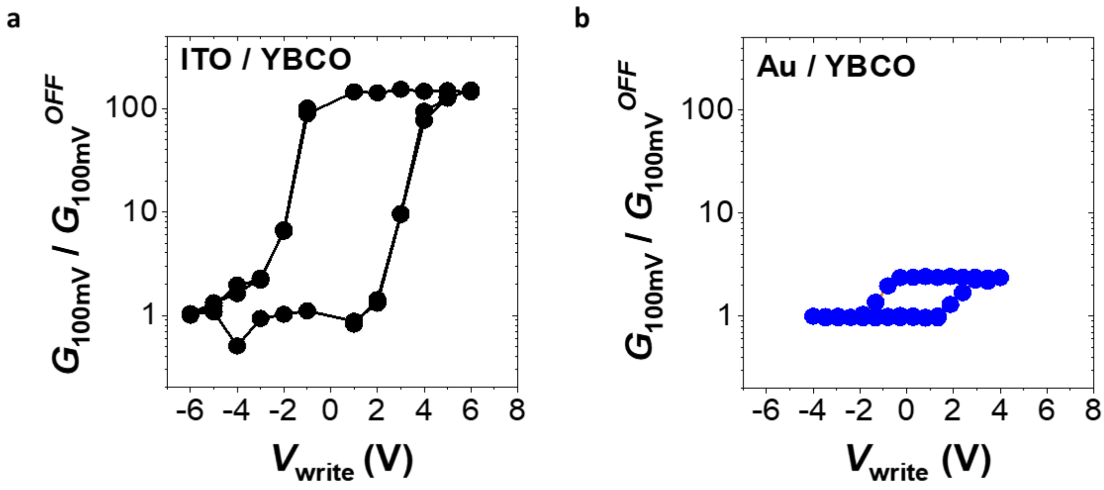

Figure S3: Comparison of conductance switching behaviours with and without reducing electrodes. Conductance switching measured at 100 mV in (a) an ITO/YBCO junction and in (b) Au/YBCO junction. One can see that the conductance changes are more than two orders of magnitude in the first case, but only a factor of ~ 2 in the second, as expected from earlier experiments in Au/YBCO contacts[6]. This very different behaviours demonstrates that a metal with a tendency to oxidize (ITO) is required to obtain large conductance switching effects, evidencing that oxygen exchange is the underlining mechanism.

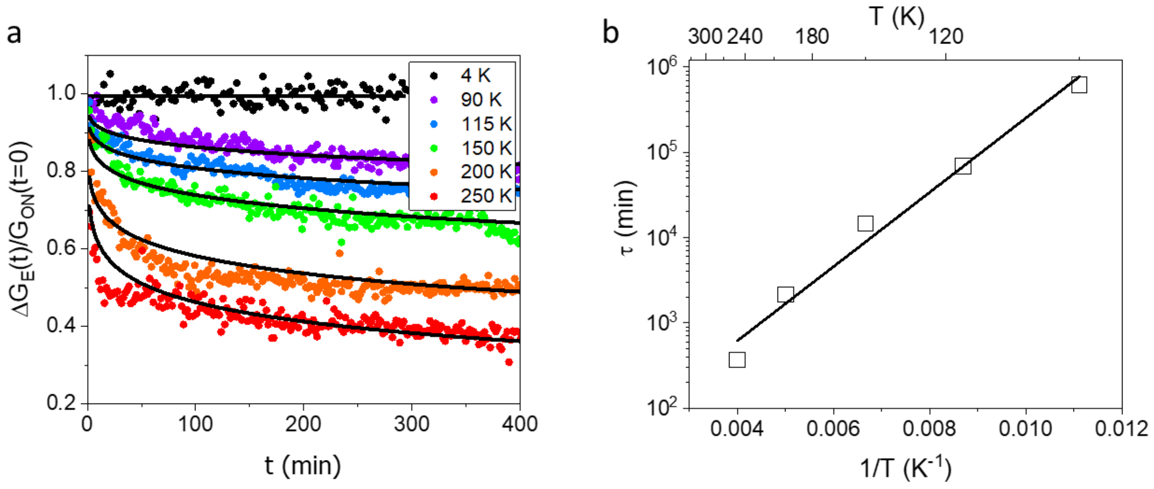

Figure S4: Relaxation of the ON state. (a) Normalized conductance switching $\Delta G_E(t)/G_{ON}(t=0)$ as a function of time after the junction was set in the ON state (V_{write} = 6 V at T = 3.2 K), measured at different temperatures T (see legend). The solid lines show the best fit to a stretched exponential $\Delta G_E(t)/G_{ON}(t=0) = e^{-(t/\tau)^\beta}$ obtained by fixing a range for $\beta = 0.21 \pm 0.1$ and leaving the time scale τ as a free parameter (similar fits are obtained if both parameters are set free, which yields $0.15 < \beta < 0.27$). **(b)** Temperature dependence of τ (hollow symbols), which is well described by $\tau \propto e^{E_a/K_BT}$ (best fit indicated by the straight line) with K_B the Boltzmann constant and $E_a = 0.09 \pm 0.01$ eV a characteristic energy scale. This $E_a \sim 0.1$ eV is well in the range of the values found in oxygen exchange reactions in YBCO[7], which further supports the redox scenario.

The long time-scale of the relaxation process as well as the stretched exponential behavior suggests that the migration of oxygen from YBCO into ITO, which is the microscopic mechanism leading to the relaxation of the ON into the OFF state, requires activation over a distribution of energy barriers. This can be understood if one considers that oxygen atoms near the interface more easily migrate to oxidize ITO than those further from it, especially as oxygen is gradually depleted from YBCO and must migrate from further in-depth the YBCO layer.

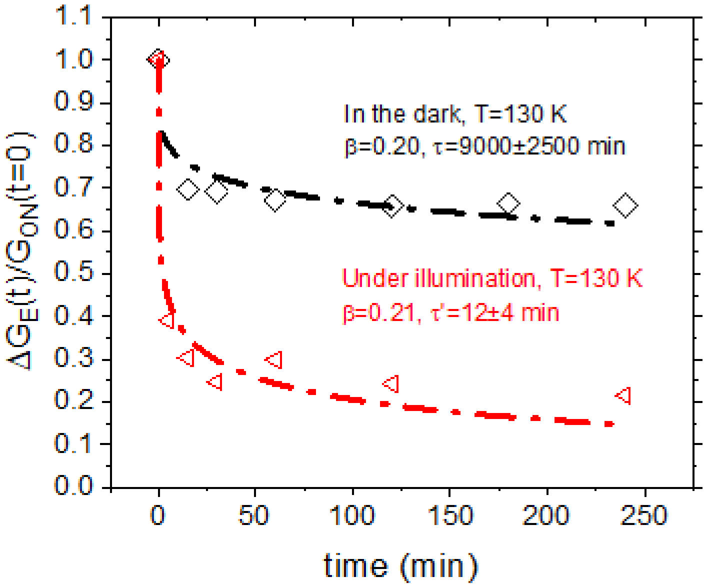

Figure S5: **Quantitative study of the illumination-accelerated relaxation in the ON state.** Normalized conductance switching $\Delta G_E(t)/G_{ON}(t=0)$ as a function of time, measured at a fixed T = 130 K in the dark and under illumination (λ = 405 nm and P= 465 mV cm^{-2}). Before each measurement, the junction was set in the ON state (V_{write} = 6 V at T = 3.2 K), then the temperature was stabilized at T = 130 K, and the conductance *vs*. time was measured (hollow symbols). The dashed lines are the best fits to a stretched exponential using the parameters displayed in the legend.

Notice that the relaxation under illumination is much faster than in the dark. Of course, that is characterized by a much shorter τ. Indeed, under illumination, the relaxation rate is equivalent to that expected at a much higher temperature. This "equivalent temperature", T_{eq} can be estimated from the stretched exponential parameters using $\Delta G_E(t)/G_{ON}(t=0) = e^{-(t/\tau)^{\beta}}$ and $\tau \propto e^{E_a/K_B T}$, which yields:

$$T_{eq} = \left(\frac{1}{T} + \frac{K_B}{\beta E_a} ln\left(\frac{\ln(\Delta G_E(t))}{\ln(\Delta G'_E i(t))}\right)\right)^{-1},$$

where the primed quantities correspond to those under illumination. Using the conductance values for 60 min < t < 250 min in Fig. S5, $\beta = 0.21$ and $E_a = 0.1$ eV (estimated from Fig S4b), we obtain T_{eq}~ 320 K-350 K. That is, the relaxation rate is roughly equivalent to that expected ~ 200 K above the actual sample temperature (which we determine by measuring the electrical resistance of the junction's YBCO electrode under illumination).

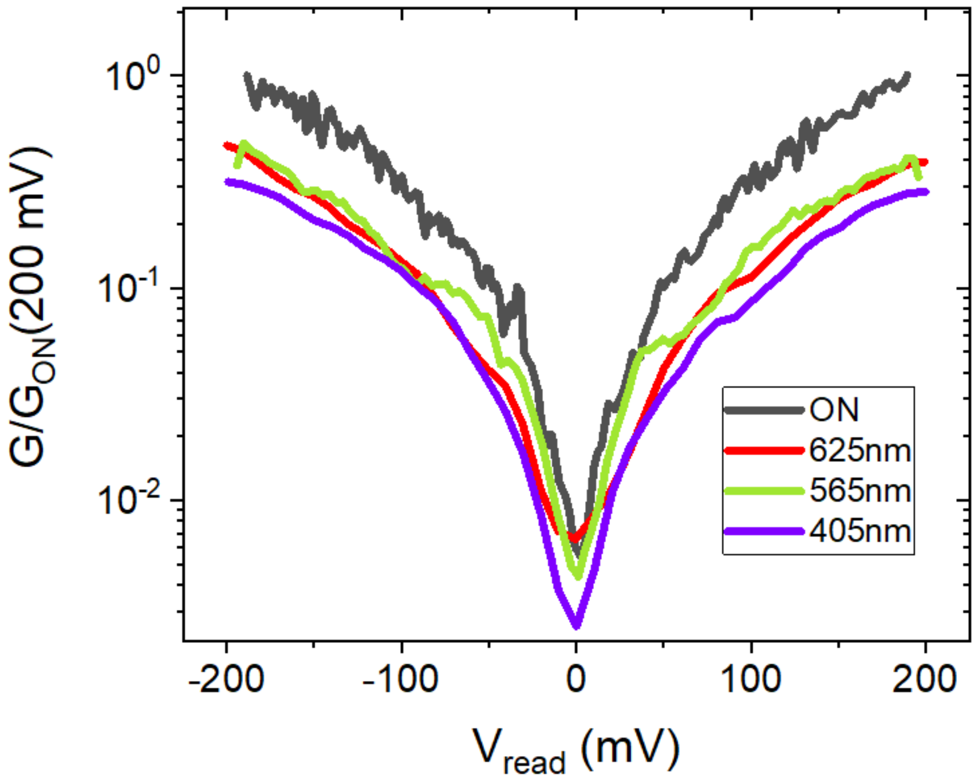

Figure S6: Light effects in the ON state as a function of wavelength. Normalized conductance *vs*. bias at 4 K for a single junction after different illuminations. The black curve is measured in the dark after having set the junction in the ON state. The colored curves correspond to the remnant states after illuminating with different wavelengths (see legend). The illumination conditions are identical for the three wavelengths: prior to each illumination round, the sample was reset to the ON state at 4 K, then illuminated at 95 K. The optical power was 200 mW cm^{-2} and the illumination time was 120 minutes in all cases.

One can see that the illumination effects are comparable for the three wavelengths, which span across the visible spectrum.

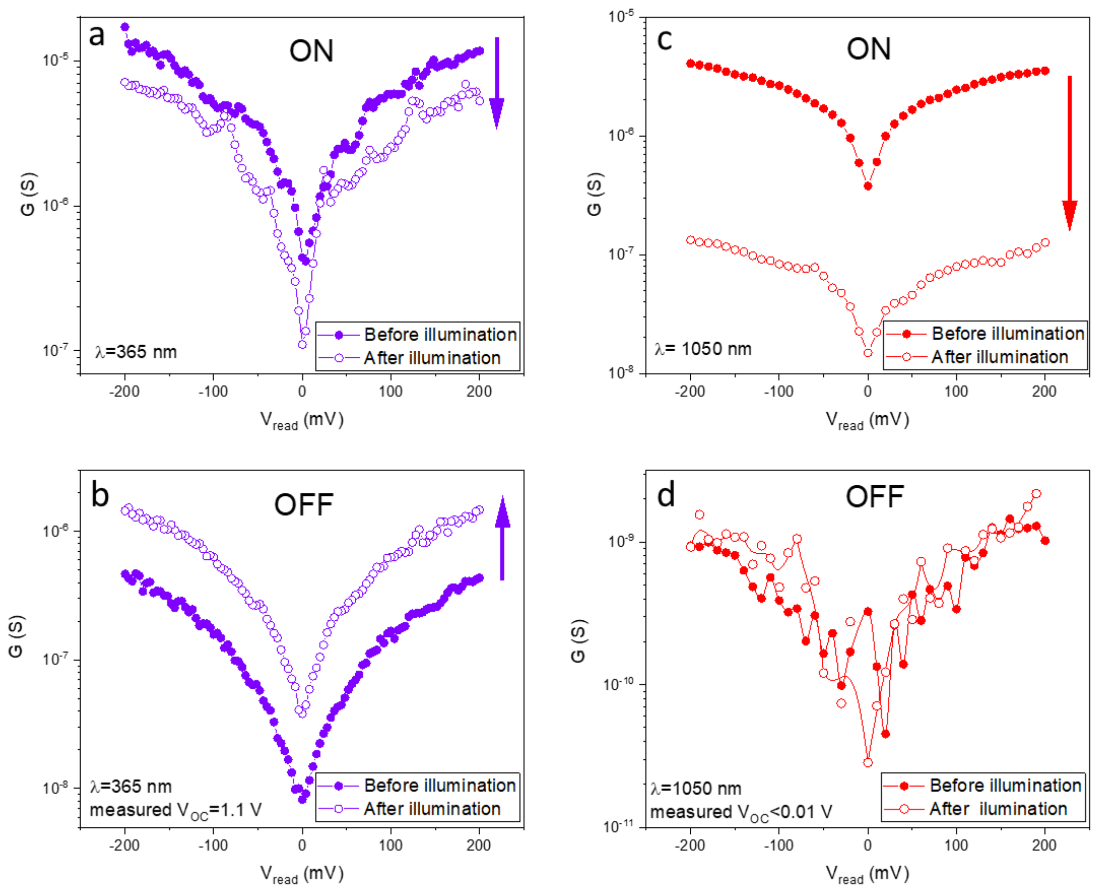

Figure S7: Comparison of UV and IR effects in the ON and OFF state. Conductance *vs.* bias at 4 K measured for two junctions in the ON and OFF states, under different illumination conductions. **(a)** and **(b)** display data for a junction illuminated with UV light (λ= 365 nm, optical power 580 mW cm^{-2}, illumination time 120 min), **(c)** and **(d)** correspond to a junction illuminated with IR light (λ = 1050 nm, optical power 121 mW cm^{-2}, illumination time 60 min). Before each illumination (at 4 K for the ON state at 95 K in the OFF state), the junctions were set in ON/OFF by applying positive/negative V_{write} at 4 K.

(a) and (b) demonstrates that UV light can produce either a nonvolatile decrease or increase of the conductance, depending on whether the illumination takes place in the ON or OFF state. At variance, and while IR light produces a very strong conductance decrease in the ON state (Fig S7 c), it causes no conductance change in the OFF state. Interestingly, this is linked to the fact that no measurable photovoltage V_{OC} is produced by IR light, which contrasts with the large V_{OC} = 1.1 V observed under UV light. The connection between V_{OC} and nonvolatile conductance changes in the OFF state is further substantiated in Fig. S8.

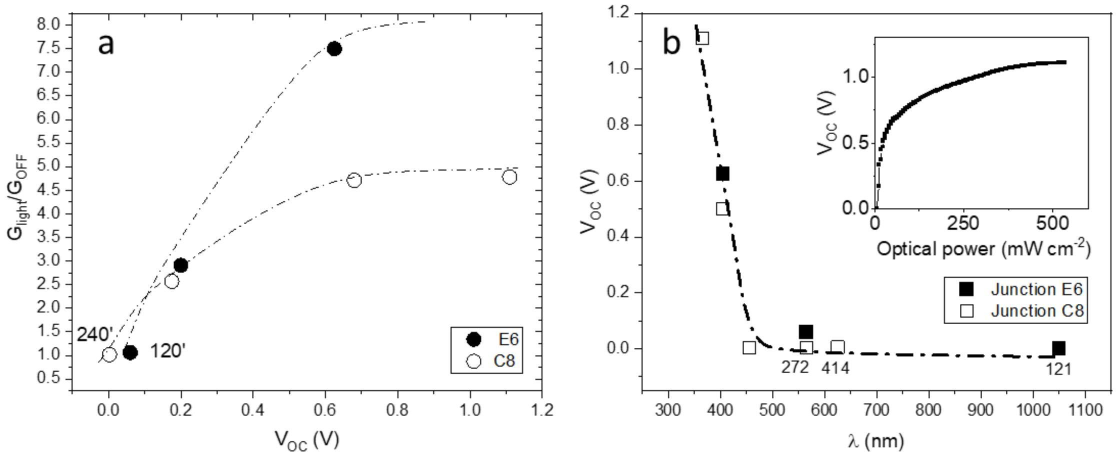

Figure S8: Correlation between photovoltage, non-volatile conductance changes and illumination wavelength in the OFF state. (a) Relative (persistent) conductance increase measured after illumination *vs*. photovoltage V_{OC} measured during illumination, in the OFF state. Displayed data correspond to two similar junctions (E6 and C8) measured under a variety of illumination conditions (wavelength and power). The illumination times are 30 min for E6 and 120 min for C8, except for the points indicated by labels (for the lower V_{OC} longer illumination times were allowed to make sure this factor is not limiting G_{light}/G_{OFF}). One can see that no persistent conductance increase is observed in the absence of photovoltage and that, for each junction, the size of the conductance enhancement scales with the size of the photovoltage -at least up to a certain threshold value of around ~ 0.7 V. This demonstrates a causal relation between photovoltage (during illumination) and persistent conductance change (after illumination). **(b)** Dependence of the measured photovoltage on the illumination wavelength. For each junction (C8 and E6), the used optical power is similar for all wavelengths (respectively 580 mW cm^{-2} for C8 and 240-270 mW cm^{-2} for E6), except for the data points in which a different power is indicated by the label (in mW cm^{-2}). The inset displays a photovoltage *vs*. optical power curve at 4 K that is typical for the junctions showing large photovoltages (the example corresponds to $\lambda = 365$ nm). This inset shows that the photovoltage tends to saturate quickly as the optical power is increased. From (b) one concludes that the photovoltage is much stronger in the UV range and drops rapidly as the wavelength is increased across the visible range.

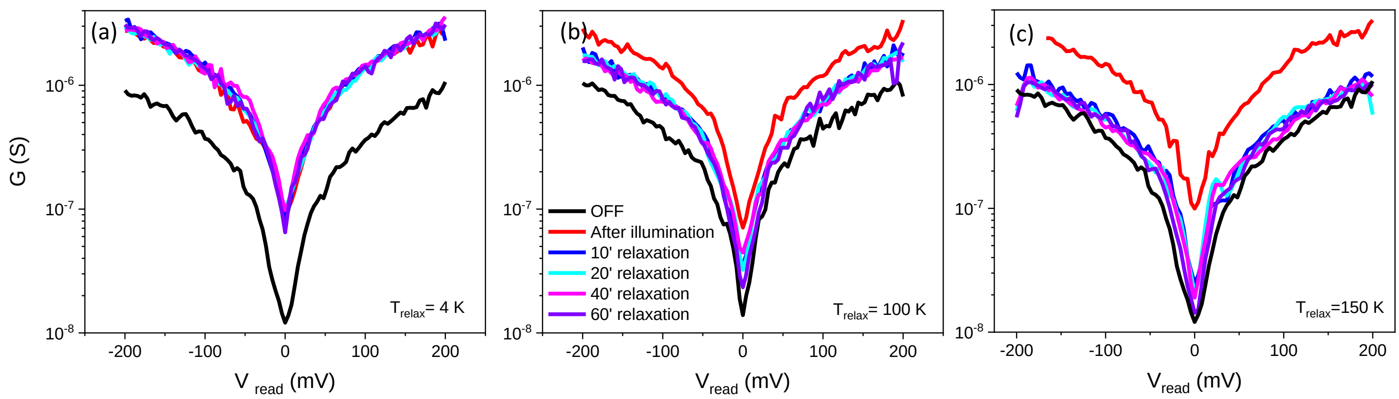

Figure S9: Relaxation after illumination Relaxation of the differential conductance at different temperatures (a) T_{relax} = 4 K, (b) T_{relax} = 100 K, and (c) T_{relax} = 150 K following illumination. All the conductance *vs.* V_{read} curves were measured at the same T = 4 K. The protocol for monitoring relaxation at different T_{relax} = 4 K is as follows. First, the OFF state is set by applying V_{write}< 0 at T = 4 K, which yields the curves labeled OFF (black). Then illumination is carried at T= 4 K, with yields the conductance state displayed by the red curve. The temperature is then set to T_{relax} at which the sample is stabilized during a given relaxation time. Subsequently, the sample is set again at T = 4 K for measuring the conductance curve (each color corresponds to a different relaxation time as indicated in the legend). This protocol allows direct comparison of the raw data for the different T_{relax} (different panels)

One can see in (a) that for T_{relax} = 4 K the conductance level is virtually nonvolatile and remains as set by illumination, regardless of the time allowed for relaxation. However, at T_{relax} = 100 K (b), the conductance relaxes halfway into the OFF state for the longest relaxation times. At 150 K (c) the conductance gets significantly closer to the OFF state. Thus, after illumination sets the conductance at some intermediate level, the junction naturally relaxes in the dark towards the OFF state at a rate that increases with increasing temperature. This is as expected from the data of the relaxation from the highest-conductance (ON) state, shown in Fig. S4.

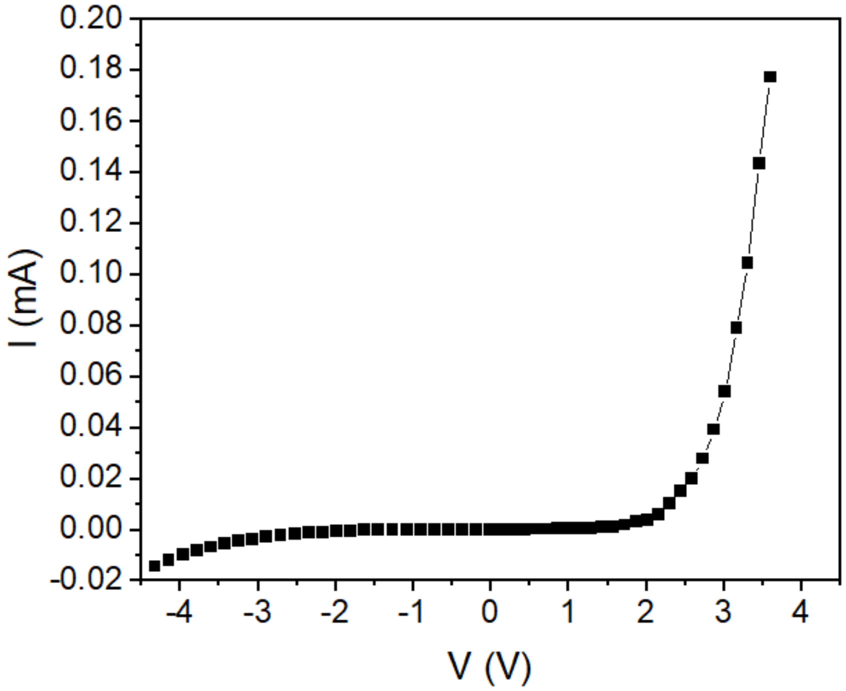

Figure S10: Evidence of diode behaviour via measurements in extended bias range. I(V) of one of the studied YBCO/ITO junctions in the OFF state, measured at 4 K. In this extended bias range, the diode behaviour characteristic of a p-n is observed.

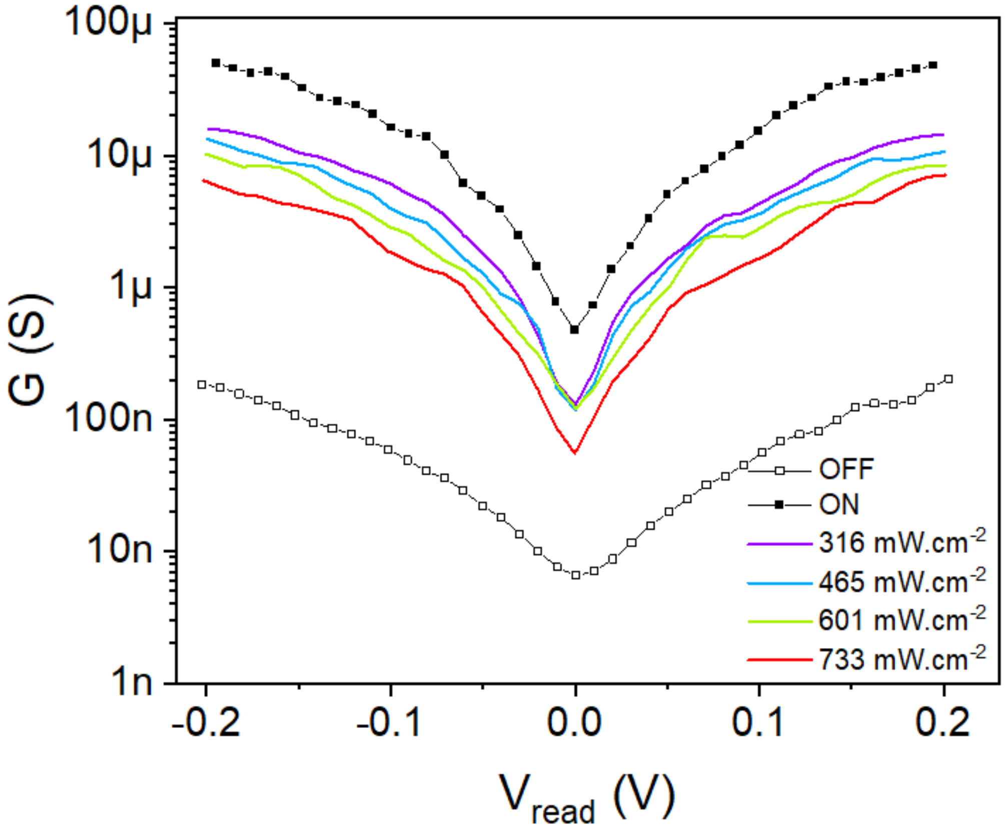

Figure S11: Illumination power dependence of photoinduced conductance decrease in the ON state. Differential conductance of a YBCO/ITO junction as a function bias, measured at 3 K, in the OFF, ON and intermediate states reached after illumination (fixed time t =120 minutes and $\lambda =$ 405 nm) with variable power (see legend). Before each illumination, the junction was set in the ON state by applying $V_{write} > 0$.

Material	Reduction reaction	Reduction potential E_0 (V)
YBCO	Cu^{+3} + e $\rightarrow Cu^{+2}$	2.4
ITO (In_2O_3 90%/SnO_2 10%)	In^{+3} +e $\rightarrow In^{+2}$	-0.49

Supplementary Table S1 Reduction potentials of the elements involved in the interfacial redox reaction. Obtained from the *Table of Standard Electrode Potentials* by Milazzo et al., edited by Willey (Chichester), 1978. For YBCO, we have considered the expected[8,9] change of valence of Cu when oxygen is removed from the structure. For ITO we select In since In_2O_3 constitutes 90% (weight) of the material. We see from the table that YBCO has the highest reduction potential. Thus, the redox reaction through which oxygen is transferred into the counter-electrode ITO is expectedly spontaneous. The voltage required to reverse this reaction equals ΔE_0, which is around 2.9 V.

Supplementary references

1. Pierret, R. F. *Semiconductor Device Fundamentals*. *New York* (Prentice Hall, 1996).
2. Ito, T., Takenaka, K. & Uchida, S. Systematic deviation from *T* -linear behavior in the in-plane resistivity of $YBa_2Cu_3O_{7-y}$: Evidence for dominant spin scattering. *Phys. Rev. Lett.* **70**, 3995–3998 (1993).
3. Kim, H. *et al.* Electrical, optical, and structural properties of indium-tin-oxide thin films for organic light-emitting devices. *J. Appl. Phys.* **86**, 6451–6461 (1999).
4. Rouco, V. *et al.* Quasiparticle tunnel electroresistance in superconducting junctions. *Nat. Commun.* **11**, 1–9 (2020).
5. Jönsson-Åkerman, B. J. *et al.* Reliability of normal-state current - Voltage characteristics as an indicator of tunnel-junction barrier quality. *Appl. Phys. Lett.* **77**, 1870–1872 (2000).
6. Plecenik, A. *et al.* Influence of bias voltage history on conductance properties of YBaCuO/normal metal junctions. *Phys. C Supercond. its Appl.* **301**, 234–242 (1998).
7. Cayado, P. *et al.* Untangling surface oxygen exchange effects in YBa 2 Cu 3 O 6+x thin films by electrical conductivity relaxation †. *Phys. Chem. Chem. Phys* **19**, 14129 (2017).
8. Temmerman, W. M., Winter, H., Szotek, Z. & Svane, A. Cu valency change induced by O doping in YBCO. *Phys. Rev. Lett.* **86**, 2435–2438 (2001).
9. Sæterli, R., Selbach, S. M., Ravindran, P., Grande, T. & Holmestad, R. Electronic structure of multiferroic BiFeO3 and related compounds: Electron energy loss spectroscopy and density functional study. *Phys. Rev. B - Condens. Matter Mater. Phys.* **82**, 29–33 (2010).
